# From skincare to watercare: Tackling salicylic acid pollution with hybrid glow plasma-cavitation technology

**DOI:** 10.1016/j.ultsonch.2025.107468

**Published:** 2025-07-22

**Authors:** Federico Verdini, Nicolò Desogus, Emanuela Calcio Gaudino, Giancarlo Cravotto

**Affiliations:** Department of Drug Science and Technology, University of Turin, via P. Giuria 9, 10125 Torino, Italy

**Keywords:** Salicylic acid, Hydrodynamic cavitation, Non-thermal plasma, Hybrid advanced oxidation processes, Wastewater treatment, Chemical dosimetry

## Abstract

Salicylic acid (SA), widely used in dermatological pharmaceuticals and as a primary metabolite of aspirin, is frequently detected in wastewater treatment plants (WWTPs) at concentrations between 3 and 900 mg/L. Given the ECHA’s predicted no-effect concentration (PNEC) of 162 mg/L for sewage treatment plants (STPs), high SA levels pose a risk to microbial communities responsible for biological oxidation. This study explores an innovative hybrid advanced oxidation process (AOP) that combines hydrodynamic cavitation (HC) and electrical discharge (ED) plasma for SA degradation, targeting environmentally friendly pretreatment before secondary biological treatment. Experiments were conducted at the pilot scale (5 L volume, 330 L/h flow rate) using model solutions with SA concentrations of 40 and 80 mg/L. While SA is typically used as a chemical dosimeter for hydroxyl radical (•OH) quantification in cavitational processes, its rapid degradation under HC/ED plasma prevents such use in this setup. For comparison, lab-scale ultrasound (US)-assisted treatments (500 kHz, 250 W, 0.1 L) were performed as a benchmark. HC/ED plasma treatment achieved over 98 % SA removal in both deionized and tap water within 20 min, whereas US treatment achieved only 54 % and 37 % removal after 120 min. Energy yield and electrical energy per order (EEO) analyses revealed that HC/ED is significantly more energy-efficient than US, particularly in tap water. Finally, the HC/ED method was evaluated through a Technology Readiness Level (TRL) assessment to estimate its scalability and applicability in real WWTP scenarios.

## Highlights

• A hybrid HC/ED plasma treatment was developed for efficient salicylic acid degradation.
• A 98 % removal of SA and its by-products was achieved in 20 min using HC/ED plasma.
• The HC/ED process demonstrated high energy efficiency and moderate EEO values at pilot scale.
• TRL evaluation confirmed the scalability of the HC/ED reactor for real wastewater use.

## Graphical abstract

**Figure:**
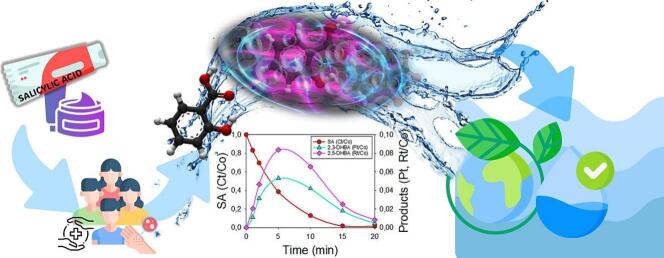

## 1 Introduction

**Fig. 1:**
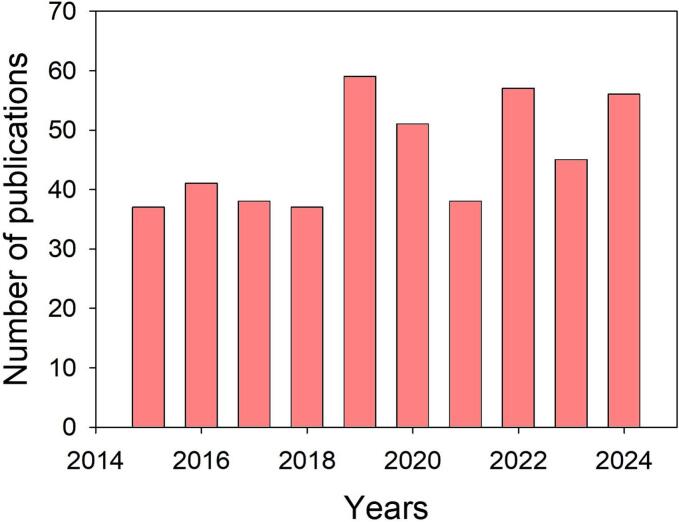
Number of published papers on SA degradation in water from 2015 to 2024 (.

Source: Web of Science. Keywords: “aspirin degradation in water” and “salicylic acid degradation in water”)

The contamination of water bodies has been threatening the health of both humans and aquatic animals in recent years, posing significant environmental risks. Although water pollution is typically associated with the improper disposal of industrial effluents, agricultural runoff, and refinery by-products into natural water sources, the extensive use of active pharmaceutical compounds (APIs) is also recognized as a major contributor to environmental contamination [1]. These substances can enter aquatic ecosystems via various pathways, including excretion, improper disposal, and insufficient removal during wastewater-treatment processes. In fact, salicylic acid (SA) is frequently detected in surface waters. SA is widely used in dermatological formulations for the treatment of skin conditions such as acne, psoriasis, calluses, corns, keratosis pilaris, and warts [2], owing to its keratolytic, antimicrobial, and anti-inflammatory properties. The global SA market size was valued at USD 547.5 million in 2024 and is expected to grow at a Compound Annual Growth Rate (CAGR) of 8.2 % from 2025 to 2030. This grow is mainly driven by the pharmaceutical sector, which accounted for the largest market share of 45.0 % in 2024, with this being primarily due to the increasing applications of SA in the development of pharmaceutical products for the treatment of the ever-higher number of cases of dermatological conditions [3]. As a direct consequence, SA has been detected in a high concentration range in WWTP inflows (3–900 mg/L) and at lower concentrations in surface and groundwater (0–10 mg/L) [4]. Additionally, the presence of SA in the environment can also be ascribed to the extensive use of acetylsalicylic acid (aspirin, ASP) for the treatment of fever, inflammation, migraines, and for reducing the risk of major adverse cardiovascular events. In fact, ASP is immediately hydrolyzed to SA in human plasma, and this is subsequently excreted with urine (as salicylates) at rates ranging from 10 % to 85 % in the presence of other metabolites, such as hydroxybenzoic acids [5]. While the European Chemicals Agency (ECHA) has classified SA as non-harmful to aquatic organisms, readily biodegradable, not potentially bioaccumulable, and not dangerous to the environment, it has also predicted a no-effect concentration (PNEC) of 162 mg/L for sewage treatment plants (STPs) [6], which may be cause for concern. Indeed, concentrations exceeding the PNEC-STP can have a negative impact on the microorganisms used in biological treatment processes, causing low treatment efficiency and incomplete contaminant removal. As previously mentioned, the sampling of several WWTP inflows has revealed several cases in which measured concentrations exceeded not only the PNEC-STP, but also the LC50 for fish (684 mg/L) and daphnids (397 mg/L), and the EC50 for green algae (325 mg/L), according to the Ecological Structure Activity Relationships (Ecosar Application 2.2) predictive model (Fig. S1) [7]. Consequently, academic interest in the degradation of SA has grown substantially over the last decade, as is clearly observable in the increasing number of scientific publications on this topic (Fig. 1). This trend highlights the rising recognition of SA as an emerging contaminant of concern, and the need for effective and sustainable degradation technologies.

**Fig. 2:**
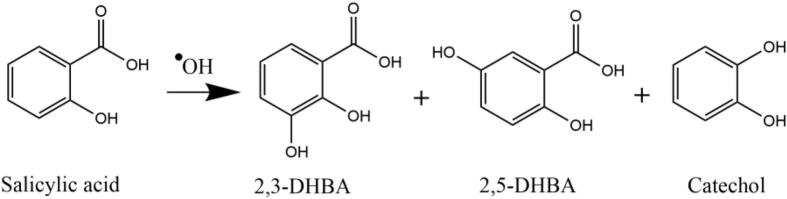
Reaction mechanism of SA dosimetry tests.

In addition, SA is commonly used as a reagent for the quantification of hydroxyl radicals (•OH) in several cavitation-based advanced oxidation processes (AOPs) [8], due the selective attack of •OH on salicylic acid to produce 2,3-dihdroxybenzoic acid (2,3-DHBA), 2,5-dihdroxybenzoic acid (2,5-DHBA) and, in some instances, catechol (Fig. 2). These reaction products can be quantified during AOPs as an indirect measurement of the •OH generated *in-situ*. However, other by-products (1,4-dihydroxybenzene, fumaric acid, maleic acid, malonic acid, oxalic acid, and acetic acid) can be identified at the end of dosimetry tests, suggesting that the unselective degradation of SA to the desired products (2,3- and 2,5-DHBA) occurs as well as their possible over degradation [9,10].

Despite extensive research into the use of AOPs for water treatment, only a few well-established systems have been developed from laboratory- to pilot-scale implementation. Moreover, the applicability of many emerging AOPs remains challenging due to the use of toxic oxidants and technologies with high energy demands [11,12]. In addition, limitations in experimental design and planning may hinder the objective assessment and scalability of novel approaches. Studies involving new oxidants, materials, technologies, and radical-generation mechanisms often lack the essential data required to evaluate performance in real-water matrices, including energy and chemical demand. For instance, reaction-rate constants are frequently reported based only on contaminant degradation time, without providing information regarding energy input, oxidant dosage, and the reactivity of the target compound. Although current studies offer useful insights into wastewater treatment at lab scale, they lack clear information on how effective and applicable these processes would be on a large scale. In this study, we have explored and compared the efficacies of acoustic cavitation (ultrasound, US) and a hybrid approach that combines hydrodynamic cavitation (HC) with electrical discharge (ED) plasma (namely non-thermal, cold or glow plasma) for the simultaneous degradation of SA (and its metabolites) without the use of external oxidizing chemicals, to evaluate their potential as promising candidates for use as sustainable pre-treatment technologies for WWTP biological secondary treatment. In fact, cavitational treatments (both US and HC) are considered environmentally friendly AOPs for WWT and are characterized by low energy consumption for the breakdown of organic pollutants towards harmless end products, including CO_2_, H_2_O, and less toxic derivatives [13]. Local variations in water pressure, either induced by the propagation of acoustic waves (US) or by the passage of liquid through a constriction (HC), can generate vaporous and gaseous cavities (cavitation bubbles) that grow and subsequently implode, releasing energy that dissociates water vapor molecules, consequently forming •OH radicals. However, although US and HC have already been extensively applied for the degradation of pharmaceuticals in water [14,15], they exhibit higher organic-pollutant mineralization rates when used in combination with other AOPs, such as photocatalysis, Fenton, H_2_O_2_, and non-thermal plasma [16]. This study presents a comparative evaluation of two cavitational AOPs for the degradation of SA in water, in the absence of any additional oxidizing agents. Specifically, a high-frequency US reactor is compared with an innovative pilot-scale hybrid system that combines HC and non-thermal ED plasma (HC/ED). In addition, critical aspects regarding the use of SA as an •OH probe have emerged during the performance evaluation of the proposed hybrid approach, whose technological maturity has been assessed via the implementation of a specific technology readiness level (TRL) scale for AOPs.

## 2 Material and Methods

### 2.1 Pilot-scale SA degradation under hybrid HC/ED plasma

**Fig. 3:**
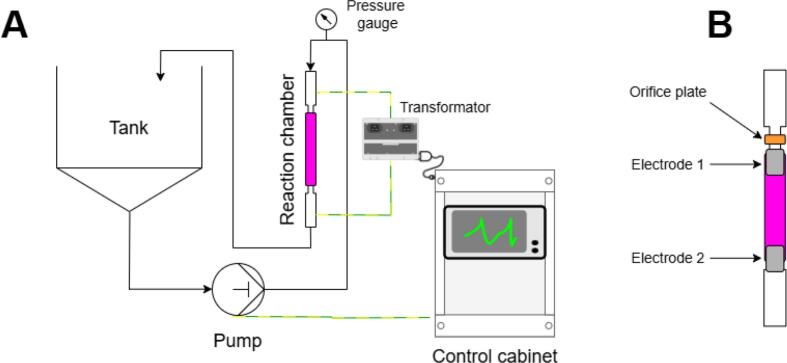
A) Schematic of the HC/ED plasma reactor. B) Detail of the reaction chamber.

Hybrid HC/ED plasma treatments were carried out in the prototype reactor schematically illustrated in Fig. 3, with a detailed description provided in a previous study [17]. HC was generated inside a quartz tube (200 x 8 mm) reaction chamber using a triplex plunger pump (SPECK Pumpen Verkaufsgesellschaft GmbH, Neunkirchen am Sand, Germany) and a 4-holed orifice plate. The propagation of ED through the cavitation bubbles for the generation of non-thermal plasma was ensured by two electrodes located at the ends of the reaction chamber. In the alternating current (AC) channel, an oscillator generates a rectangular signal with a frequency of 49 kHz and delivers it to the electrodes. The duty cycle (D) was set at 70 % (default parameter) and the consequent operative pulse width (T_ON_) was 14 µs, calculated according to equations (1), (2).

(1) $$T_{\mathrm{TOT}}=\frac{1}{f[kHz]}=\frac{1}{49kHz}=0.020ms=20\mu s$$

(2) $$T_{\mathrm{ON}}=\frac{D\cdot T_{\mathrm{TOT}}\left[\mu s\right]}{100}=\frac{70\cdot 20\mu s}{100}=14\mu s$$

For each test, 5 L of contaminated solution (with initial SA concentrations of 40 and 80 mg/L) were recirculated within the reaction chamber and the working pressure was set at 20 bar, with a corresponding measured flow rate of 330 L/h, for a total time of 20 min (Table 1).

**Table 1:** Operation parameters of HC/ED plasma degradation treatments.

Inlet pressure (bar)	Flow rate (L/h)	t_R_ (min) a	n° of cycles a
20	330	0.04	22

a Residence time and number of cycles for 20 min of recirculation treatment.

The treated water temperature was kept constant at 20 ± 2 °C using a chiller unit (DLSB-5/10, Zhengzhou Keda Machinery, and Instrument Equipment Co., Ltd., Zhenghou, Henan, China) that was connected to a heat exchanger embedded in the 30 L stainless-steel reservoir tank. The energy consumption of the entire HC/ED plasma system was displayed on its control panel, while the energy consumption of the chilling system was calculated using a wattage meter (PM230 brennenstuhl®, Tübingen, Germany). A schematic overview of the system components and operational steps employed to evaluate the potential use of the HC/ED plasma reactor as pre-treatment technology for WWTP biological secondary treatment is represented in Fig. S2.

### 2.2 Lab-scale SA degradation under US

The US-assisted degradation tests of all target contaminants were performed in an US system composed of a US generator (Weber Ultrasonics AG, Karlsbad, Germany) and a stainless-steel ultrasonic tank with a surface of 0.056 m^2^ equipped with three piezo transducers. All batch experiments were carried out by filling the US tank with 3 L of deionized water and performed at a fixed US power of 250 W at a frequency of 500 kHz, on a 0.1 L solution of contaminated water placed in an Erlenmeyer 0.5 L flask submerged in the US tank. Under these operating conditions, the fixed US surface power and volume densities were 4.5 kW/m^2^ and 81 kW/m^3^, respectively. An additional coiled heat exchanger, connected to a chiller unit set at 8 °C, was placed in the US tank to keep the temperature constant at 20 ± 1°C. A blade agitator (set at 400 rpm), submerged in the contaminated solution, was used to enhance the mass transfer in selected experiments. A further SA degradation experiment was carried out at a US frequency of 120 kHz, without changing the other parameters.

### 2.3 HPLC analysis

The treated samples (both under US or HD/ED) were directly analyzed on a high-performance liquid chromatography system (Waters Corp., Milford, USA) equipped with a 2998 photodiode array (PDA) detector (UV/DAD, Waters Corp., Milford, USA). A reversed phase 4.6 mm x 150 mm Kinetex C18 (Phenomenex) column, with 5 μm particles, was used for the separation of analytical compounds. The mobile phase was CH_3_CN/TFA (0.1 %)–H_2_O/TFA (0.1 %), and gradient mode was used (Table S1) at a flow rate of 1.0 mL/min. The injection volume was 20 μL. The best selected wavelength for SA and 2,5-DHBA was 237 nm, while for 2,3-DHBA it was 247 nm.

### 2.4 GC–MS analysis

In order to perform a qualitative investigation into the by-products generated during the HC/ED plasma treatments, 25 mL of each collected sample was freeze-dried for 24 h at a temperature of −80 °C and at a pressure of 80 mbar (Telstar Lyotest, Azbil Telstar SL, Terrassa, Spain). The samples were re-dissolved into 0.5 mL of ultrapure water acidified with 1 M HCl before proceeding with liquid–liquid extraction using 1 mL of chloroform (CHCl_3_), which was subsequently recovered, with the addition of 25 μL of *N,O*-*bis*(trimethylsilyl)trifluoroacetamide (BSTFA) under heating to 50 °C for 1 h for the derivatization of the analytes. An additional liquid–liquid extraction was carried out on the residual water phase with 1 mL of ethyl acetate. The so-treated samples were injected into the GC–MS instrument equipped with an Agilent 6890 gas chromatograph (Agilent Technologies, USA) and an HP-5 capillary column (length: 30 m, internal diameter: 0.25 mm, and film thickness: 0.25 μm) coupled to an Agilent Network 5973 mass detector. The initial oven temperature was set at 50 °C and increased to 300 °C over 10 min.

## 3 Results and discussion

### 3.1 SA degradation under hybrid HC/ED plasma

**Fig. 4:**
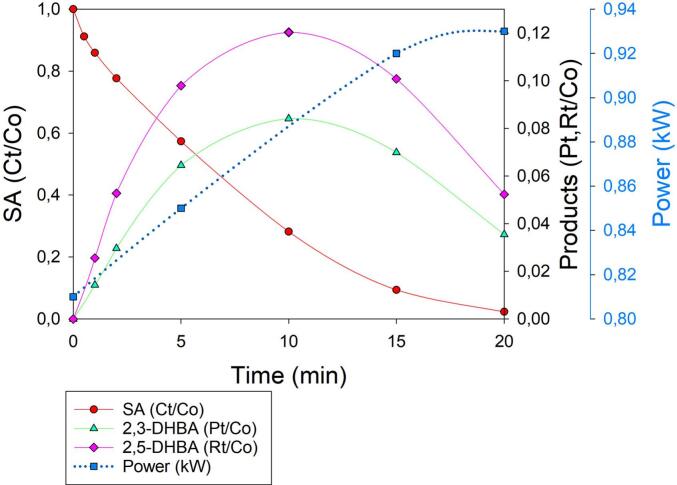
SA degradation under HC/ED plasma in deionized water. C_0_ = 40 mg/L. Concentration values of SA, 2,3-DHBA and 2,5-DHBA are normalized to the SA initial concentration.

Due to its widespread use in personal care and dermatological products, salicylic acid (SA) is often found to be present in surface and wastewater at moderate-to-high concentrations [4]. Its persistence in aquatic environments, combined with its potential ecotoxicological effects, underscores the need for effective removal strategies. In this context, the present study focuses on investigating the degradation of SA using an innovative hybrid approach that is based on HC coupled with electrical discharge plasma (ED). This combined technique aims to enhance the generation of reactive species and improve degradation efficiency, offering a potentially effective solution for the treatment of recalcitrant contaminants, such as SA. Due to the high-energy-electrons avalanche that occurs in plasma reactors, ED plasma exhibits very high pollutant removal and mineralization efficiency, driven by the formation of highly reactive oxidizing species, without the need for the very oxidizing chemicals commonly deployed in AOPs. Generally, an alternating current is applied between two electrodes surrounded by an inert gas, such as argon, nitrogen, helium, and oxygen, or by a mixture of them, in contact with polluted water [18]. The impact of ED electrons with both gas and water molecules promotes the generation of plasma, composed of highly reactive species such as •OH, •H, •O, O_2_•^−^, O_3_, and H_2_O_2_, and UV light generated by hydrolysis, ionization, and vibrationally excited molecules [19]. ED plasma has recently been coupled with HC with the aim of: (1) improving the limited chemical effects (i.e., generation of •OH) of HC; (2) scaling up ED plasma technology to pilot and industrial scales (a task that remains a challenging endeavor without coupling with HC); and (3) improving the overall efficiency of wastewater treatment, especially in terms of mineralization rate [20]. Therefore, a first SA degradation experiment was carried out in which 200 mg of SA was dissolved in 5 L of deionized water (C_0_ = 40 mg/L), which was characterized by a conductivity of 3 µS/cm and a pH of 6.5. The SA solution was subsequently recirculated inside the HC/ED plasma reactor (flow rate of 330 L/h) working at 20 bar, and an ED frequency and pulse width of 49 kHz and 14 µs, respectively. Besides monitoring the concentration of SA (C_t_) during the treatment, the concentrations of both 2,3-DHBA (P_t_) and 2,5-DHBA (R_t_), the two main degradation by-products, were also monitored. As reported in Fig. 4, 20 min of HC/ED plasma treatment provided quantitative SA degradation (C_t_/C_0_ > 0.0232; final Ct concentration < 1 mg/L; degradation rate > 98 %). As expected, the oxidative degradation of SA leads to the formation of 2,3- and 2,5-DHBA due to •OH attack on the aromatic ring. The by-products are simultaneously generated and reach their highest concentration (3.5 and 5.2 mg/L of 2,3- and 2,5-DHBA, respectively) after 10 min of treatment, with a higher selectivity towards 2,5-DHBA. At longer treatment times, the concentration of both by-products decreased despite the continued presence of residual SA, suggesting that their possible simultaneous formation and degradation occur. This trend may indicate that once a threshold concentration of hydroxylated intermediates is reached, further exposure to reactive species, such as hydroxyl radicals or other plasma-generated oxidants, leads to their breakdown into smaller, possibly less toxic or more biodegradable compounds. Moreover, the concurrent presence of SA and its intermediates may result in competitive reactions for reactive species, influencing both the degradation pathway and the kinetics of transformation products. These observations support the existence of a dynamic equilibrium between formation and consumption processes under prolonged treatment conditions. However, after 20 min, these by-products were not completely degraded (P_t_, R_t_/C_0_ < 0.05), underlying the need for a monitoring of the concentration of oxidative by-products during AOPs, rather than focusing solely on the starting pollutant.

**Fig. 5:**
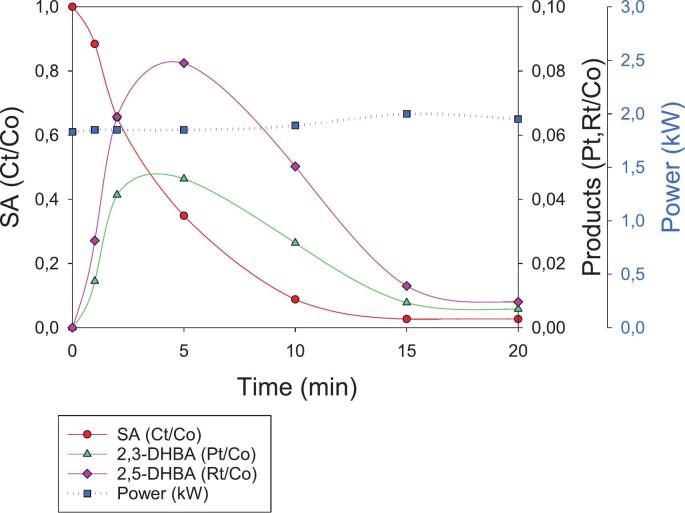
SA degradation under HC/ED plasma in tap water. C_0_ = 40 mg/L. Concentration values of SA, 2,3-DHBA and 2,5-DHBA are normalized to initial SA concentration.

Additionally, the power of the HC/ED plasma reactor was monitored and increased from 0.81 kW (t = 0 min) to 0.93 kW (t = 20 min) (Fig. 4). The power variation during treatment can be ascribed to the scavenging effect of SA, which quickly reacts with •OH, modifying the equilibrium conditions between the propagation of the ED through the cavitation bubbles and the usual rate of •OH formation. The scavenger effect of SA was confirmed by the variation in ED plasma color from its usual bright pink purple to blue, which is a phenomenon that has already been observed in a previous study [21], where the degradation of a diuretic (furosemide) was carried out in the presence of typical •OH scavengers, such as ethanol and *tert*-butyl alcohol. Moreover, the increase in power and the progressive restoring of plasma color (under normal conditions) during treatment are in line with a decrease in SA concentration, limiting scavenger activity. Under these operative conditions, the average consumed power of the HC/ED plasma reactor was 0.88 kW, which, combined with the measured power of the chiller unit (0.08 kW) and then further normalized to the total volume treated in 1 h (0.3 m^3^), allowed the electrical energy dose (EED) of the process to be calculated at 3.2 kWh/m^3^. To approach real aqueous sample-treatment conditions, the same experiment was repeated by dissolving the same amount of SA in 5 L of tap water, which was characterized by a pH of 7.1 and a conductivity value of 391 µS/cm (Table S2). This significantly improved ED propagation, bringing the entire system back to a more stable state both in terms of visuals (no plasma color variations) and supplied power, which remained constant at ∼ 1.90 kW throughout treatment. Using tap water, quantitative SA degradation (C_t_/C_0_ > 0.0268) was achieved after only 15 min (Fig. 5), whereas 20 min was required in the previous experiment (Fig. 4). The highest concentrations of 2,3-DHBA (1.7 mg/L) and 2,5-DHBA (3.1 mg/L) were observed after only 5 min and their quantitative degradation (P_t_, R_t_/C_0_ < 0.008) was achieved at the end of the treatment, underlying the higher stability and efficiency of the ED plasma generated in tap water. Despite the negative effect that an increase in water conductivity had on •OH production in ED plasma-assisted processes [22], higher electron density and temperature can lead to the higher formation of secondary species (such as H_2_O_2_, O_3_,•O, O_2_^–^), which enhanced SA degradation. Nevertheless, the higher power of the HC/ED system observed in tap water (∼1.90 kW), over deionized water (0.88 kW on average), increased the EED of the process to 7.3 kWh/m^3^.

**Fig. 6:**
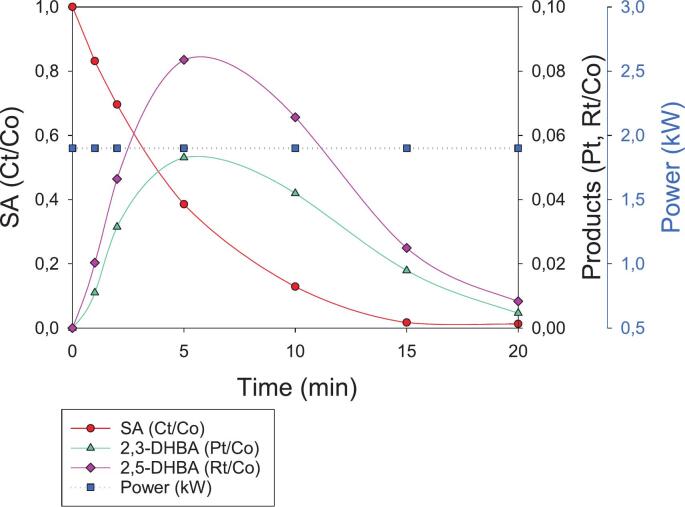
SA degradation under HC/ED plasma in tap water. C_0_ = 80 mg/L. Concentration values of SA, 2,3-DHBA and 2,5-DHBA are normalized to initial SA concentration.

The comparison of the two experimental kinetic rate constants, calculated according to a pseudo-first order kinetic model, confirmed that the test carried out in tap water provided faster degradation (k = 0.2466 min^−1^; R^2^ = 0.9981) than that in deionized water (k = 0.1772 min^−1^; R^2^ = 0.9707). More details are reported in Table 2 and Fig. S3. For the sake of comparison, a further experiment was carried out under HC alone (ED plasma “off”) and SA degradation was observed to be negligible (C_t_/C_0_ = 0.8731) after 20 min of treatment (Fig. S4). A further additional test was carried out in which the SA starting concentration was increased to 80 mg/L to evaluate the efficiency of the HC/ED plasma reactor in treating very concentrated polluted water. As shown in Fig. 6, SA and by-products were quantitatively degraded (C_t_/C_0_ > 0.017; P_t_, R_t_/C_0_ > 0.025) in only 15 min of treatment, as observed in the previous experiment.

**Table 2:** Statistical parameters of the nonlinear regression model applied to the SA degradation test carried out in deionized and tap water under HC/ED plasma.

Water source	SA C_0_ (mg/L)	k (min^−1^)	SE (k)	Adjusted R^2^	SEE	F-test (ANOVA)
Deionized water	40	0.1772	0.0126	0,9658	0.2505	170.5360 (P < 0,0001)
Tap water	40	0.2466	0.0054	0,9981	0.0716	1981.8373 (P < 0,0001)
Tap water	80	0.2364	0.0175	0.9681	0,3286	186.3376 (P < 0,0001)

SE: standard error of k (P-value < 0.0001); SEE: standard error of estimate. (Software: *SigmaPlot 15.0.0.13*; Equation model: f = y0 + a*x).

The trends of the curves representing the C_t_/C_o_ and P_t_,R_t_/Co ratios from the two experiments conducted at 40 and 80 mg/L are comparable (Fig. S5), as expected, as they correspond to a pseudo first-order kinetic model that is independent of pollutant concentration. Consequently, the kinetic rate constant for the experiment conducted with the 80 mg/L SA solution (0.2364 min^–^1) was comparable to that of the experiment conducted with the 40 mg/L solution (0.2466 min^–^1) (Table 1).

The evaluation of the energy yield of the processes, calculated as indicated in equation (3), and showed in Table 3) further confirmed that the hybrid HC/ED plasma reactor was capable of degrading higher amount of pollutant with the same EED (7.3 kWh/m^3^), demonstrating the high potential of the application of this new hybrid approach to water reclamation processes. The experiment carried out in deionized water showed the highest energy yield (754 mg/kWh). However, the instability of ED plasma propagation in deionized water evidenced by a linear increase in ED power instead of the constant value observed in tap water, limits the representativeness of this scenario for real-world water treatment cases. Consequently, the EED values calculated in deionized water cannot be considered fully optimized or entirely representative of the actual potential of this treatment.

(3) $$\mathrm{Energy}\;\mathrm{yield}\;\left[mg\cdot {\mathrm{kWh}}^{-1}\right]=\frac{\mathrm{Degraded}\;\mathrm{SA}\;[mg]}{\mathrm{Energy}\;\mathrm{consumption}\;[kWh]}$$

To the best of our knowledge, no studies have reported the degradation of salicylic acid (SA) using non-thermal plasma technologies, either alone or in combination with other advanced oxidation processes (AOPs). As a result, a direct comparison with similar plasma-based approaches is not possible at this stage. However, a broader discussion comparing the degradation efficiency of the present method with other AOPs reported in the literature will be provided in the following sections. As an attempt to further investigate the formation of SA by-products during HC/ED plasma treatment and to compare the results with those available in literature, the treated samples were concentrated 25 times and analyzed by GC–MS. According to Zhe *et al.* [23] the oxidation of SA by means of combined UV/O_3_ AOP leads to the formation of several and consecutive by-products, such as 2,3- and 2,5-DHBA, catechol, phenol, short-chain alcohols, diols, and acids, such as lactic, succinic, fumaric and oxalic acids. The GC–MS analyses (section 2.4) on samples treated for 5 and 10 min, starting from an initial SA concentration of 80 mg/L, confirmed the formation of catechol (traces), lactic acid, 1,2-propanediol, 1,4-butanediol, and 1-butanol during the HC/ED plasma-assisted experiments (Fig. S6). An additional by-product, identified as benzoic acid, was detected in trace amounts during this study. To the best of our knowledge, this compound has not been previously reported as a degradation product of salicylic acid under AOP conditions (Fig. S6C). A complete understanding of the formation mechanism for this by-product is highly challenging due to the complex state of matter (cold plasma) in which the degradation occurred. Possible mechanisms may involve a selective –OH reduction due to the presence of ^•^H, typically generated in plasma [24] (Fig. S7), and/or direct C-O bond cleavage, as observed by Vickie Pan *et al.* [25], during the assessment of the plasma dissociation reaction kinetics in plasma-assisted methyl methacrylate polymerization. However, the GC–MS analysis carried out on the sample obtained at the end of the treatment (Fig. S6D) demonstrated the complete degradation of SA and its associated oxidation by-products in only 20 min, corroborating the findings presented in section 3.1. These results are particularly significant from an ecotoxicological perspective, demonstrating the very high efficiency of the presented technology in the simultaneous degradation of SA and toxic by-products, such as catechol and phenol.

**Table 3:** Energy parameters of SA degradation treatment under HC/ED plasma.

Water source	SA C_0_ (mg/L)	Degraded SA (mg) a	Energy consumption (kWh) a	Energy yield (mg/kWh) a	EED (kWh/m^3^) ^b^
Deionized water	40	181	0.24	754	3.2
Tap water	40	195	0.55	355	7.3
Tap water	80	393	0.55	715	7.3

a Values calculated for 15 min of treatment ^b^ Values normalized to 60 min of treatment.

#### 3.1.1 SA chemical dosimetry test: Advantages and critical aspects

As established in Section 1, the most common chemical dosimeter for the quantification of •OH in AOPs is salicylate/salicylic acid [8]. SA offers several advantages [26]: (1) the hydroxylated products only derive from •OH attack to the aromatic ring (high specificity); (2) the easy separation and detection of hydroxylated products by HPLC; and (3) the kinetics of SA hydroxylation are favored compared to other dosimeters such as Fricke, iodide dosimetry (Weissler) or terephthalate [27]. However, optimal SA concentration and pH rely on the •OH production technology. In fact, Amin *et al.* [28] have optimized SA concentration in a venturi HC reactor, finding that 750 mg/L yields the highest radical formation. They also investigated the effect of several operative parameters, such as upstream pressure and constriction shape, and observed a linear increase in •OH production. However, the authors discovered that high SA concentrations led to several anomalies in cavitation behavior. At 300 mg/L, cavitation became less dynamic, with stable bubbles persisting longer in the Venturi constriction with weaker bubble collapse dynamics occurring due to the reduced water surface tension, which affects bubble nucleation and collapse, making bubbles more stable. A linear trend in •OH generation under HC has also been observed by Arrojo *et al.* [29] who carried out experiments at acidic pH (=3) to ensure that the acidic form of SA (the hydrophobic form) (∼48 %) is localized at the hydrophobic gas–liquid interface of cavitation bubbles, thereby facilitating its attack by •OH radicals generated in the same region. Based on the results achieved, presented in section 3.1, the SA dosimeter did not appear to be ideally suited for quantifying •OH radical formation in the hybrid HC/ED plasma reactor under the applied operative conditions. Indeed, during the HC/ED plasma treatment of SA, 2,3- and 2,5-DHBA were degraded after their maximal production (Fig. 4,5 and 6), while SA remained available for further •OH attacks. In contrast, Weissler chemical dosimetry, previously applied in the same reactor, allowed us to observe a linear increase in •OH concentration as a function of treatment time, proving that it is a better option for this HC/ED technology [30].

#### 3.1.2 Pilot-scale HC/ED plasma prototype reactor: Technology Readiness Level (TRL) evaluation

The technological maturity of a system can generally be measured using the Technology Readiness Level (TRL); a universal scale adopted by organizations such as NASA [31] and the European Union [32]. However, proposed TRL scales are based on general criteria for the TRL assessment of different processes (on a scale ranging from 1 to 9), without a specific framework for AOPs. Due to this lack of standardization, Hübner *et al.* [33] have attempted to establish some guidelines for systematic future research on AOPs, especially defining additional requirements for TRL advancement. They proposed to: (1) evaluate the stability of the technology material in water; (2) compare the new technology with a reference process; (3) determine the effective generation of oxidative species and the electric energy per order (EEO) as a standardized energy consumption value. The EEO was proposed by Bolton *et al.* [34] in 1996 and then included in an IUPAC technical report [35] in 2001, and considers the electric energy required (kW) to decrease the concentration of a target contaminant by one order of magnitude (90 %), as function of treated volume (m^3^), for AOPs in which contaminant decay follows pseudo-first order kinetics (Equation (4)).

(4) $$\mathrm{EEO}\left[kWh\cdot m^{-3}\cdot {\mathrm{order}}^{-1}\right]=\frac{P\left[kW\right]\cdot t\left[h\right]}{V\left[m^{3}\right]\cdot log\frac{C_{o}}{C_{f}}}$$

where “*P”* is the power (kW), “*t”* the expected treatment time (h) to achieve 90 % degradation (extrapolated from the kinetic model), “*V”* the volume of treated water and “*C*_0_” and *“C*_f_” are the initial and final concentration of the contaminant, respectively (set to express 90 % degradation).

**Fig. 7:**
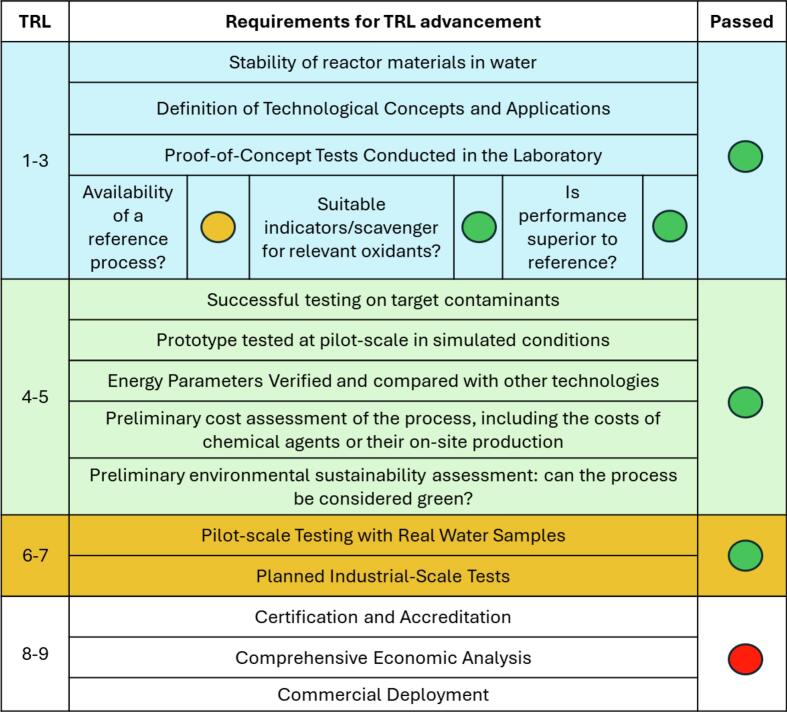
Proposed and implemented TRL scale for the evaluation of pilot-scale HC/ED plasma prototype reactor maturity using a traffic light coding system.

To assess the maturity of the pilot-scale HC/ED plasma prototype reactor exploited in this work, the general TRL scale has been implemented with the incorporation of the specific requirements for AOPs, as proposed in Fig. 7.

Despite the absence of real reference processes for the presented HC/ED plasma technology in the literature, only similar technologies have been compared, prompting the use of the orange indicator. In fact, despite the few literature articles advocating the use of hybrid HC and ED plasma technologies [36], the described systems differ significantly from the presented pilot-scale prototype, especially in terms of design, scale (volumetric capacity), ED plasma power and frequency, geometry, and treated contaminants. However, the identification of proper chemical dosimetry for •OH quantification (i.e. Weissler dosimetry, as discussed in section 3.1.1), together with optical emission spectroscopy (OES) (Fig. S7), and the demonstrated superior performance to the “reference” [21,30] satisfy TRL requirements 1–3 (green indicator). With respect to the evaluation of energy parameters, as required for TRL 4–5, the EEO of the presented HC/ED plasma treatment for SA degradation in water has been calculated and compared to other AOPs (Table 4). After a thorough collection of AOP EEO values, Miklos *et al.* [37] have defined the AOPs characterized by EEO < 1 kWh·m^−3^ ·order^-1^ as the most readily applicable full-scale processes. Technologies with median EEOs > 1 kWh·m^−3^ ·order^-1^ are less likely to be used in typical large-scale installations in the near-term as current configurations are too energy intensive. According to the authors, these technologies require further exploration, particularly in enhancing energy efficiency. Nonetheless, AOPs with median EEO values, between 1 and 100 kWh·m^−3^ ·order^-1^, may still provide attractive solutions for specific challenges, especially in real-world applications. As shown in Table 3, the EEO for SA degradation in deionized water (2.67 kWh·m^−3^ ·order^-1^) was lower than that observed for the processes carried out in tap water (5.73 and 5.76 kWh·m^−3^ ·order^-1^ for SA C_0_ of 40 and 80 mg/L, respectively). According to Miklos *et al.* [37], these EEO values are not sufficient to consider the technology ready for industrial-scale application. However, in our view, an evaluation of potential TRL advancement to full-scale application should be complimented by additional parameters, such as energy yield (mg/kWh), operative and chemical (i.e. H_2_O_2_, O_3_, chlorine, persulfate etc.) costs, and environmental sustainability (chemical efficiency), rather than evaluating EEO alone, whose value for the same technology can vary as a result of the contaminant, its concentration and the water matrix treated. In addition, the presented HC/ED plasma technology demonstrated high degradation efficiencies with moderate EEO values without the use of chemicals and without generating waste, which is in line with the latest trends in developing green and sustainable technologies for chemical-process intensification [38]. Based on the tests carried out on the pilot-scale HC/ED plasma prototype, a new industrial-scale reactor has been developed (Fig. S8) and will be tested in the near future working with larger volumes (> 30 L) at flow rate > 2000 L/h.

**Table 4:** Comparison of EEO values calculated in this work with available literature data.

**AOP**	**EEO (kWh·m^−3^ ·order^-1^)**	**Contaminant (concentration)**	**Ref**
UV/catalyst	335.00	NA	[39]
Microwave	543.00		
Ultrasound	2616.00		
Ozonation	0.14		
O_3_/H_2_O_2_	0.20		
Electron Beam	0.33		
UV/chlorine	0.39		
O_3_/UV	0.70		
UV/persulfate	0.67		
UV/H_2_O_2_	0.78		
Photo-Fenton	2.60		
ED Plasma	3.30		
Electrochemical	38.06		
O_3_/UV	1.50	Monouron (29.5 mg/L)	[40]
TiO_2_/UV	20.00	Monouron (29.5 mg/L)	
O_3_/UV	3.00	Diuron (29.5 mg/L)	
TiO_2_/UV	25.00	Diuron (29.5 mg/L)	
Photocatalysis	80.00	Carbamazepine	[41]
O_3_/UV	79.00		
ED Plasma	14.00	Meprobamate (275 ng/L)	[42]
	5.80	Primidone (124 ng/L)	
**HC/ED plasma**	2.67	Salicylic acid (40 mg/L, deion. water)	This work
	5.73	Salicylic acid (40 mg/L, tap water)	
	5.76	Salicylic acid (80 mg/L, tap water)	
	8.79	Rhodamine B (5 mg/L, tap water)	[43]
	17.90	Tetracycline HCl (25 mg/L, tap water)	[17]

### 3.2 SA degradation under US

**Fig. 8:**
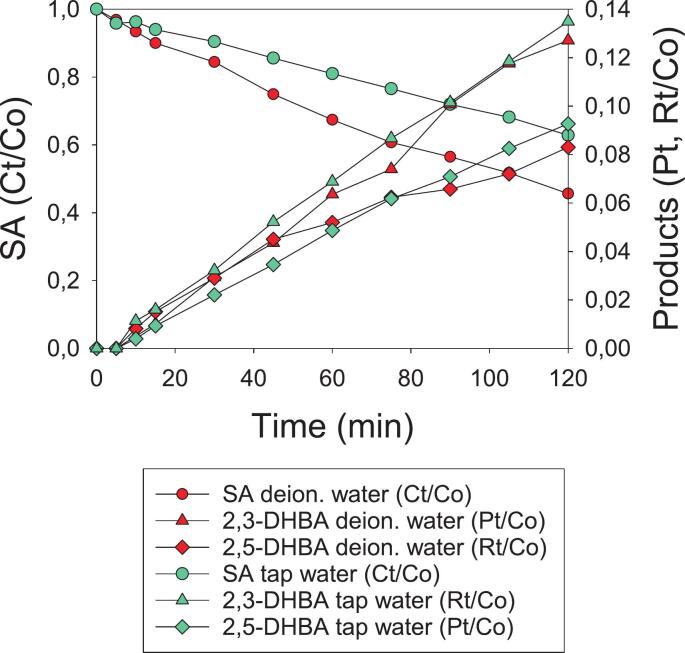
SA degradation under US in deionized (●) and tap water (●). C_0_ = 80 mg/L. Concentration values of SA, 2,3-DHBA and 2,5-DHBA are normalized to initial SA concentration.

In order to investigate another environmentally friendly AOP (i.e. without oxidizing chemicals) and to compare it with the innovative HC/ED plasma approach, two different degradation treatments of SA were also carried out under acoustic cavitation at a frequency of 500 kHz and a power of 250 W. Two different experiments were carried out on two contaminated solutions with an initial SA concentration of 80 mg/L in deionized and tap water in order to provide a comparison between US efficiency and the HC/ED plasma technology. As reported in Fig. 8, both treatments required prolonged treatment times to achieve moderate SA degradation. In fact, after 120 min of treatment, only 54 % (Ct/Co = 0.4564) and 37 % (Ct/Co = 0.6287) of the initial SA was degraded in deionized and tap water, respectively. Unlike observations under HC/ED plasma (section 3.1), US-assisted SA degradation followed zero-order kinetics, and tap water had a negative impact on degradation efficiency.

Indeed, the calculated reaction rate constants were 2.6980·10^-6^ mol·L^-1^·min^−1^ and 1.7594·10^-6^ mol·L^-1^·min^−1^ in deionized and tap water, respectively (Table 5).

**Table 5:** Statistical parameters of the nonlinear regression model applied to SA degradation tests carried out in deionized and tap water under US.

Water source	SA C_0_ (mg/L)	k (mol·L^-1^·min^−1^)	SE (k)	P-value (k)	Adjusted R^2^	SEE	F-test (ANOVA)
Deionized water	80	2.6980·10^-6^	9.3551·10^-8^	<0,0001	0.9881	1,2559·10^-5^	7275.3336 (P < 0,0001)
Tap water	80	1.7594·10^-6^	3.1297·10^-8^	<0,0001	0.9968	4.2015·10^-6^	3160.4914 (P < 0,0001)

SE: standard error of k; SEE: standard error of estimate. (Software: *SigmaPlot 15.0.0.13*; Equation model: f = y0 + a*x).

The decrease in SA degradation in tap water under US may be ascribed to several factors:

1. the presence of dissolved salts decreases gas solubility, leading to less energetic cavitation-bubble implosion (vaporous cavitation) and consequent lower •OH production. Additionally, salts increase solution viscosity leading to changes in the number of chemically active bubbles [44,45].
2. Some inorganic anions, such as carbonate and bicarbonate, can act as •OH scavengers, reducing concentrations during treatment (Table S2) [46]
3. tap water generally presents buffered pH due to the presence of bicarbonates and other buffering systems. A pH that is different to that of deionized water may alter the chemical speciation of salicylate, affecting its reactivity with •OH radicals.

Although the differences observed depend on the aqueous matrix used, the US-assisted degradation process generally demonstrated very low efficiency in contaminant removal. Additionally, to compare the EEO parameter of HC/ED plasma-assisted treatments (*section 3.1.3*) with those of US, it was first necessary to calculate the electric energy per mass (EEM), which is the appropriate parameter for zero-order degradation processes and was subsequently converted to EEO according to equations (5), (6).

(5) $$\mathrm{EEM}\left[kWh\cdot {\mathrm{Kg}}^{-1}\right]=\frac{P\left[kW\right]\cdot t\left[h\right]}{V\left[m^{3}\right]\cdot \Delta C\left[Kg\cdot m^{-3}\right]}$$

(6) $$EEO\left[kWh\cdot m^{-3}\cdot {\mathrm{order}}^{-1}\right]=EEM\left[kWh\cdot {\mathrm{Kg}}^{-1}\right]\cdot \Delta C\left[Kg\cdot m^{-3}\right]$$

where ΔC was set to express 90 % of degradation and t corresponds to the time (h) required to achieve 90 % degradation (extrapolated from the kinetic model).

Overall, this evaluation of the energy parameters (Table 6) further demonstrated the technique’s limited scalability and potential application at industrial levels, in accordance with the TRL scale (*section 3.1.3*), especially when compared to the HC/ED plasma technology.

**Table 6:** Energy parameters of SA-degradation treatment under US. C_0_ = 80 mg/L.

Water source	Degraded SA (mg) a	Energy consumption (kWh) a,^b^	Energy yield (mg/kWh) a	EED (kWh/m^3^) ^c^	EEM (kWh/Kg)	EEO (kWh/m^3^·order)
Deionized water	4.3	1.6	2.7	8000	389	28,036
Tap water	3.0	1.6	1.9	8000	582	41,920

a 120 minutes of treatment; ^b^ Energy consumption by US generator (0.4 kWh for 120 min) and chiller unit (1.2 kWh for 120 min); ^c^ Value normalized to 60 min of treatment.

Considering the potential use of a pilot-scale ultrasonic unit (Fig. S9) capable of operating in flow-through or recirculation modes at a frequency of 120 kHz and flow rate of 30 L/min and acknowledging the possibility of reduced chemical effects at this frequency, a preliminary SA degradation test was carried out at lab-scale using the previously described set-up. Frequency was lowered from 500 to 120 kHz (no intermediate frequencies could be used due to US generator characteristics). Considering the lack of SA degradation observed during the lab-scale experiment (Fig. S10), no further tests were carried out at the pilot-scale.

### 3.3 Positioning hybrid HC/ED plasma treatments of SA within the AOP Landscape

As summarized in Table 7, the main AOPs tested in the degradation of SA are photocatalysis and ozonation, used alone or in combination to increase degradation yield. A significant proportion of degradation treatments are carried out in batch mode on low starting-pollutant concentrations (1 – 30 mg/L). Despite this, only few tests achieved quantitative SA degradation (> 90 °C) when photocatalysis was combined with H_2_O_2_, and the treatment time was 90 min [49]. Ozonation treatments [50,51] led to moderate degradation yields (50 – 86 %), although experiment times were brief, being in the range of 1 to 10 min. However, batch AOPs should be translated into flow-through or recirculation systems to enable their implementation in existing WWTPs. Moderate-high SA degradations (> 79 %) were achieved in recirculation systems [47] at a high initial SA concentration (165 mg/L) using electro-Fenton and anodic oxidation (AO) combined with H_2_O_2_ electro-generation. However, although complete SA removal was achieved in the electro-Fenton AOP in 60 min, the required acidic pH (pH = 3) for Fenton-based treatments poses a challenge for practical applications in real-world water purification. Considering the findings presented in this study, the HC/ED plasma technology achieved quantitative SA degradation (> 98 %) both in deionized and tap water in very short treatment times (20 min) with moderate-high starting SA concentrations (40 and 80 mg/L), compared to US and literature data.

**Table 7:** Comparison of available literature data on SA degradation with this work.

AOP	Catalyst (loading)	SA Co (mg/L)	pH	Volume (L)	Batch	Recirculation (Flow rate)	Time (min)	%	Ref.
Photocatalysis	TiO_2_ (0.25 g/L)	165	3	3	X	✓ (200 L/h)	360	14	[47]
Anodic oxidation (AO) with electro-generated H_2_O_2_ (AO-H_2_O_2_)	X						360	79	
AO-H_2_O_2_ + Photocatalysis	TiO_2_ (0.25 g/L)						360	84	
Electro Fenton	Fe^2+^ (0.50 mM)						60	100	
Photocatalysis	Polypyrrole–TiO_2_ (1 g/L)	10	NA	0.1	✓	X	120	48.6	[48]
UV/O_2_	X	27.6	4	NA	✓	X	90	22	[49]
Photocatalysis/H_2_O_2_	N-TiO_2_ (1 g/L)						90	93–97	
	(Fe,N)-TiO_2_ (1 g/L)						90	93–97	
	P-25 TiO_2_ (1 g/L)						90	99	
Ozonation	O_3_ (1 mg/L)	1	4	NA	✓	X	0.17	95	[50]
	O_3_ (1 mg/L)	1	7,8,10				1	60–80	
Ozonation	O_3_ (2.4 mg/L)	5	4	1	✓	X	1	> 50	[51]
	O_3_ (2.4 mg/L)	10					1	> 50	
	O_3_ (2.4 mg/L)	20					10	86.2	
UV	−	10					NA	0	
UV/O_3_	O_3_ (2.4 mg/L)	5					1	> 50	
	O_3_ (2.4 mg/L)	10					10	>55.4	
Photocatalysis + O_2_	P25-TiO_2_ (0.1 g/L)	276	6.5	1	✓	X	180	> 85	[52]
Photocatalysis + O_2_ + US (20 kHz, 110 W/L)	P25-TiO_2_ (0.1 g/L)						180	> 85	
HC/ED plasma	X	40 (deion. water)	6.5	5	X	✓ (330 L/h)	20	> 98	This work
		40 (tap water)	7.3						
		80 (tap water)	7.3						
US	X	80 (deion. water)	6.6	0.1	✓	X	120	54	
		80 (tap water)	7.3				120	37	

## 4 Conclusions

The present study has investigated the efficiency of an innovative pilot-scale reactor for the degradation of SA under hybrid HC and ED plasma. In detail, 5 L tap water solutions, characterized by starting SA concentrations of 40 and 80 mg/L, were recirculated inside the reactor and the quantitative removal (> 98 %) of both SA and related oxidation by-products (2,3- and 2,5-DHBA) was achieved in only 20 min of hybrid treatment, under an inlet pressure of 20 bar and an ED plasma operative pulse width of 14 µs, following pseudo-first order kinetics. By contrast, the degradation test carried out on SA dissolved in deionized water led to the complete degradation of SA only, despite a starting concentration of 40 mg/L, with this result mainly being due to low water conductivity and consequent suboptimal ED propagation within cavitation bubbles. The calculated EEO (5.76 kWh·m^−3^ ·order^-1^) and energy efficiency (715 mg/kWh) for the treatments conducted in tap water, with an initial SA concentration of 80 mg/L, confirmed the high performance of the reactor despite the •OH scavenging behavior of SA, which is commonly used as a chemical dosimeter for •OH quantification in AOPs, but is not suitable for the technology presented here due to the extremely fast degradation of the very 2,3- and 2,5-DHBA, generated by interactions with •OH radicals, that would be used for •OH quantification. According to a proposed new TRL scale, specific for AOPs, the HC/ED plasma reactor can be categorized to a TRL of 7, especially considering industrial-scale tests planned in the near future. Lab-scale US treatment at 500 kHz and 250 W required very extended treatment times (120 min) to achieve only 54 % and 37 % SA degradation in deionized and tap water, respectively, with very high EEOs (28,036 and 41,920 kWh·m-3 ·order-1), revealing the inefficiency of US compared with HC/ED plasma. Additionally, the zero-order kinetics observed under US confirm the formation of several different oxidizing compounds, indicating that there are a number of distinct degradation mechanisms in action. In conclusion, the HC/ED plasma reactor has been used to achieve complete SA removal at moderate-high starting concentrations, with a moderate EEO, at high energy efficiency, and without requiring external chemicals or catalysts. This confirms this technology’s potential for implementation in existing WWTPs, following the successful testing of the industrial-scale version of the reactor, in order to mitigate the possible toxic effects of SA on the microorganisms used in WWTP biological treatment.

## Declaration of Generative AI and AI-assisted technologies in the writing process

During the preparation of this work the author(s) used ChatGPT in order to improve the readability and language of the manuscript and not to analyse and draw insights from data as part of the research process. After using this tool/service, the author(s) reviewed and edited the content as needed and take(s) full responsibility for the content of the published article.

## CRediT authorship contribution statement

**Federico Verdini:** Writing – original draft, Investigation, Data curation, Conceptualization. **Nicolò Desogus:** Investigation, Formal analysis, Data curation. **Emanuela Calcio Gaudino:** Writing – original draft, Validation, Supervision, Methodology, Data curation, Conceptualization. **Giancarlo Cravotto:** Writing – review & editing, Validation, Supervision, Conceptualization.

## Declaration of competing interest

The authors declare that they have no known competing financial interests or personal relationships that could have appeared to influence the work reported in this paper.
