# Supplementary material for: From skincare to watercare: Tackling salicylic acid pollution with hybrid glow plasma-cavitation technology
Source: Ultrason Sonochem. 2025 Jul 22;120:107468. doi: 10.1016/j.ultsonch.2025.107468 (PMC12312108; doi:10.1016/j.ultsonch.2025.107468)
Supplement: Supplementary Data 1 [file mmc1.docx]

**Supplementary Material**

**From Skincare to Watercare: Tackling Salicylic Acid Pollution with Hybrid Glow Plasma-Cavitation Technology**

Federico Verdini^a^, Nicolò Desogus^a^, Emanuela Calcio Gaudino^a^*, Giancarlo Cravotto^a^*

^a^ Department of Drug Science and Technology, University of Turin, via P. Giuria 9, 10125, Torino, (Italy)

* Corresponding authors. E-mail: [emanuela.calcio@unito.it](mailto:emanuela.calcio@unito.it); [giancarlo.cravotto@unito.it](mailto:giancarlo.cravotto@unito.it)


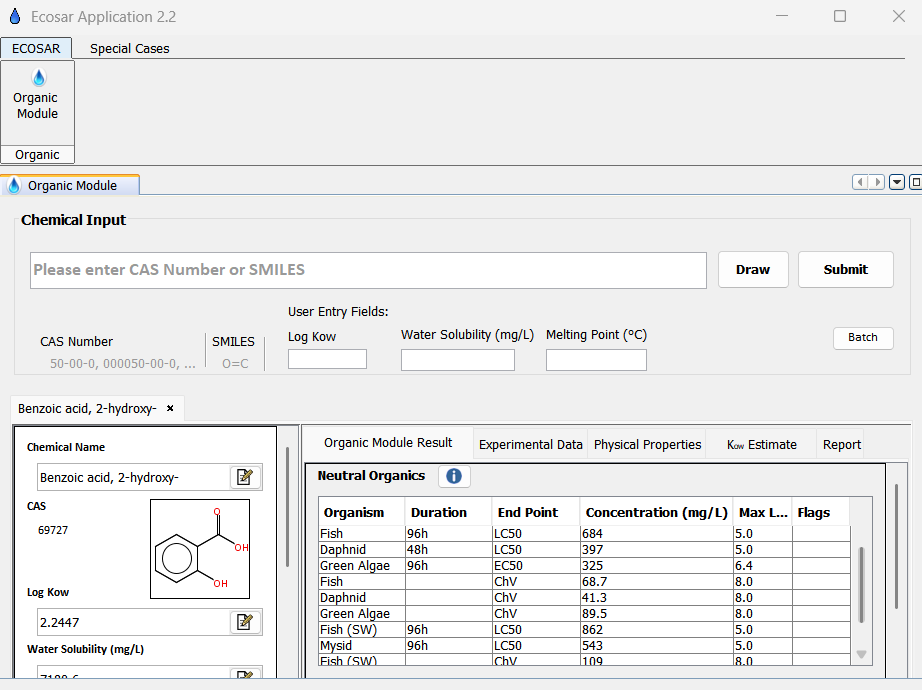


**Figure S1**. Prediction of salicylic acid ecotoxicity by Ecosar Application 2.2

| Time (min) | Flow (mL/min) | %A (H_2_O/TFA 0.1%) | %B (MeCN/TFA 0.1%) |
| --- | --- | --- | --- |
| 0 | 1 | 95 | 5 |
| 1 |  | 95 | 5 |
| 22 |  | 0 | 100 |
| 26 |  | 0 | 100 |

**Table S1**. HPLC gradient used for SA, 2,3- and 2,5-DHBA quantification


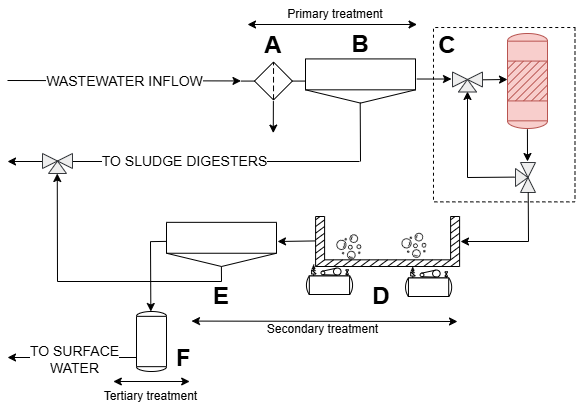


**Figure S2.** Schematic of the HC/ED plasma technology for the potential implementation in existing WWTPs (A: debris and grit removal, B: Primary clarification, C: HC/ED plasma reactor, D: biological oxidation, E: secondary clarification, F: disinfection)

| Parameter | Unit of Measurement | Average Value | Limit D.Lgs. 18/23 |
| --- | --- | --- | --- |
| Aluminum | µg/l | 49 | 200 |
| Ammonium | mg/l | <0.04 | 0.50 |
| Total Pesticides | µg/l | 0.01 | 0.50 |
| Calcium | mg/l | 55.3 | Not specified |
| Total Organic Carbon | µg/l | 656 | Not specified |
| Chlorite | mg/l | 0.14 | 0.25 or 0.70 based on disinfection method |
| Free Residual Chlorine | mg/l | 0.10 | Not specified |
| Chlorides | mg/l | 19 | 250 |
| Electrical Conductivity at 20°C | µS/cm | 391 | 2500 |
| Chromium | µg/l | 1 | 50 |
| Total Hardness | °F | 18 | Not specified |
| Iron | µg/l | 11 | 200 |
| Magnesium | mg/l | 10.8 | Not specified |
| Manganese | µg/l | 3 | 50 |
| Nickel | µg/l | 1 | 20 |
| Nitrates | mg/l | 13 | 50 |
| pH | pH | 7.7 | ≥ 6.5 and ≤ 9.5 |
| Lead | µg/l | <1 | 10 |
| Potassium | mg/l | 2.0 | Not specified |
| Residual Solids at 180°C | mg/l | 285 | Not specified |
| Selenium | µg/l | <1 | 20 |
| Sodium | mg/l | <10 | 200 |
| Sulfates | mg/l | 39 | 250 |

**Table S2.** Analysis of tap-water used in the degradation experiments carried out by SMAT - Società Metropolitana Acque Torino S.p.A.

**Figure S3.** Linearization of HC/ED plasma experimental data

**Figure S4.** Results of SA degradation under HC alone

**Figure S5.** Comparison of experimental data obtained under HC/ED plasma with SA starting concentration of 40 and 80 mg/L in tap water.

| 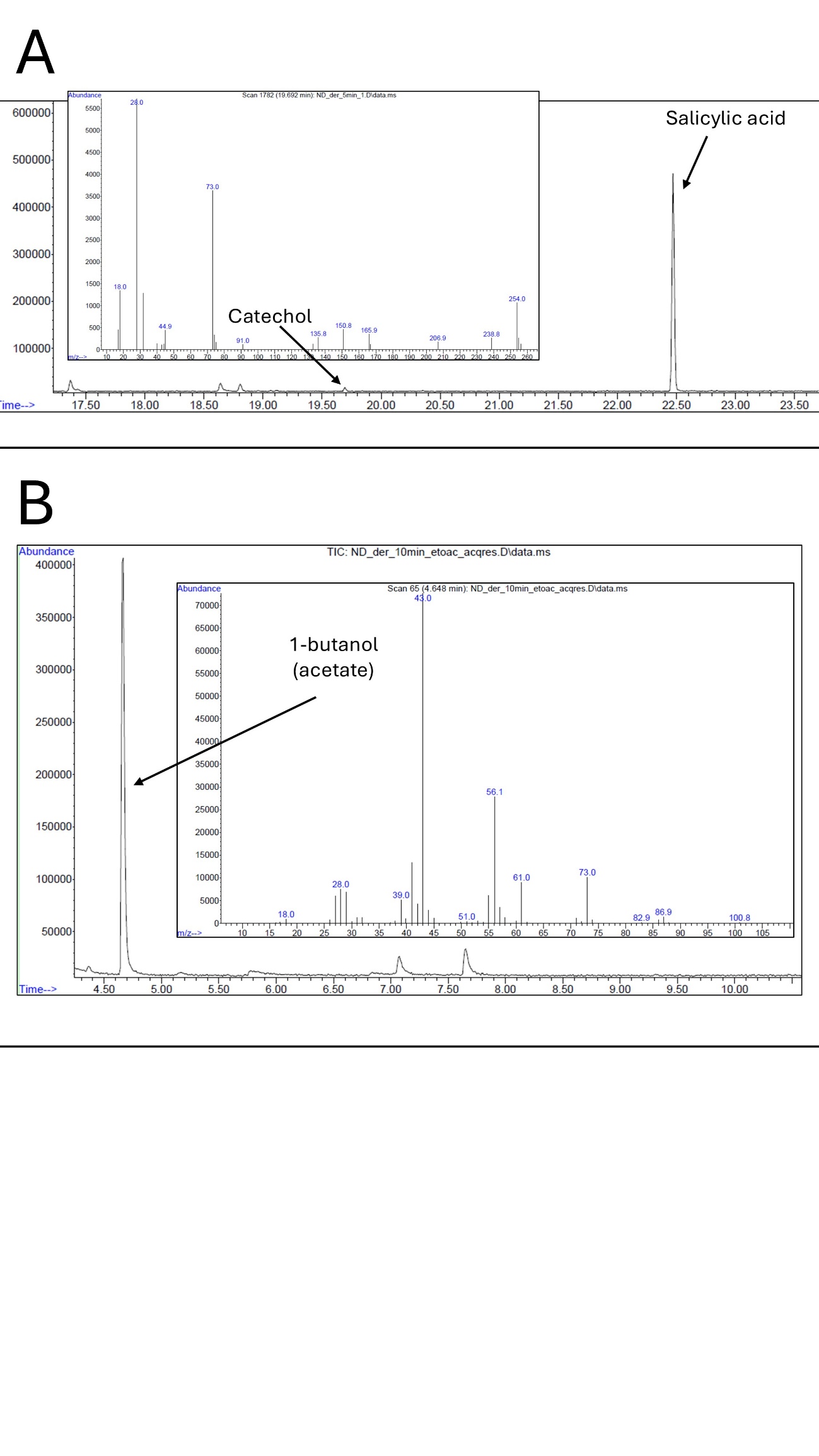 |
| --- |
| 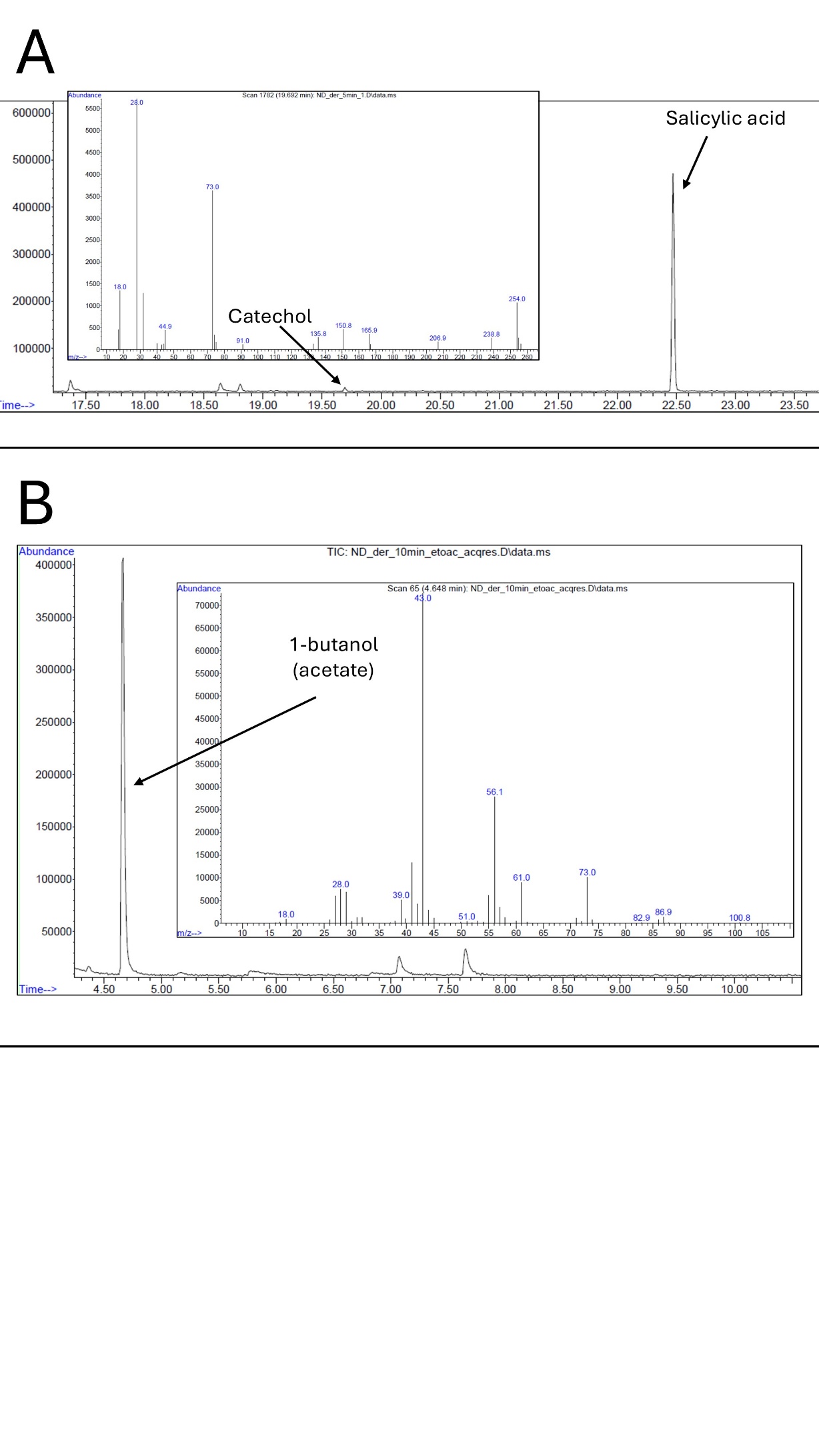 |
| 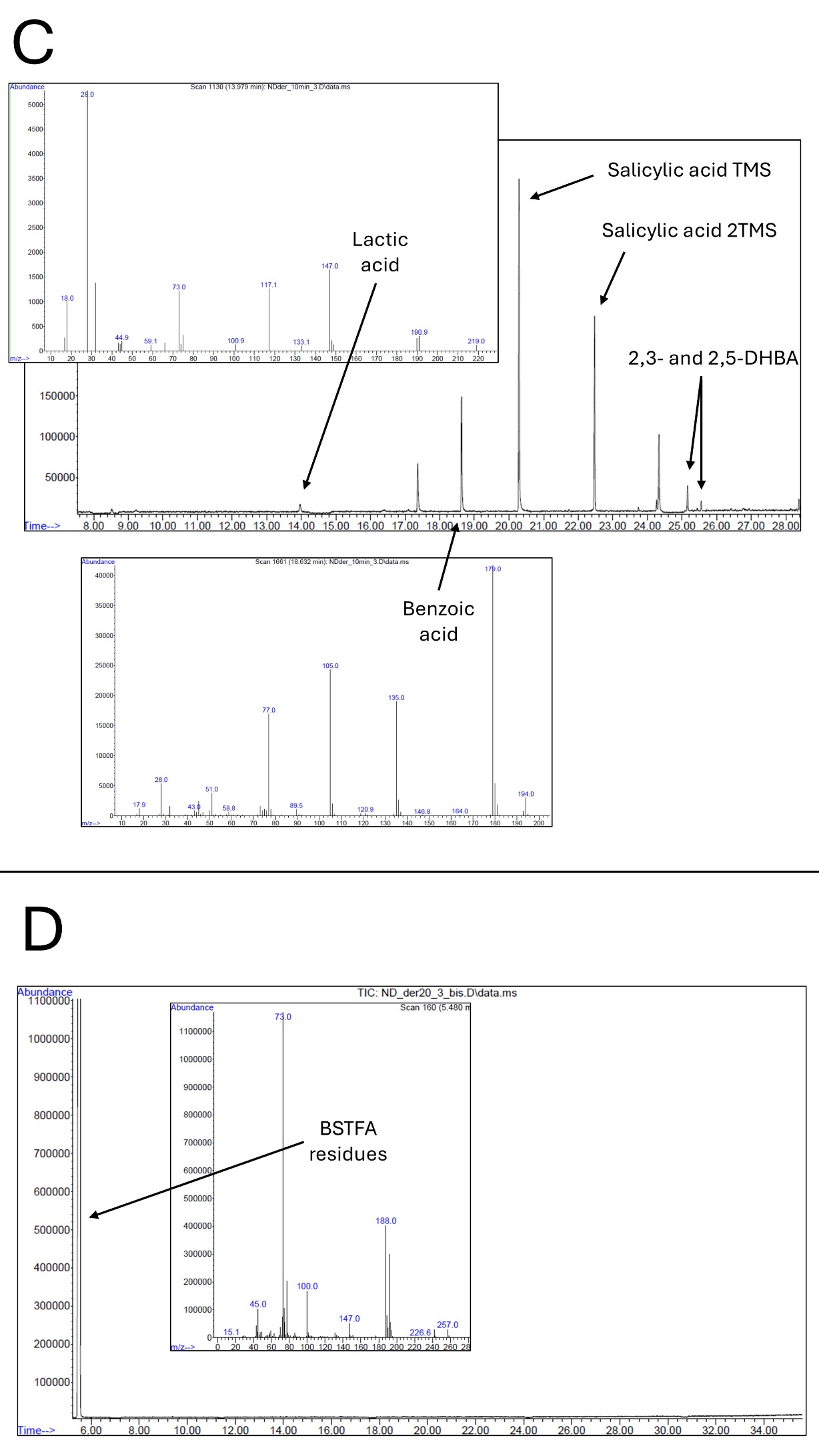 |
| 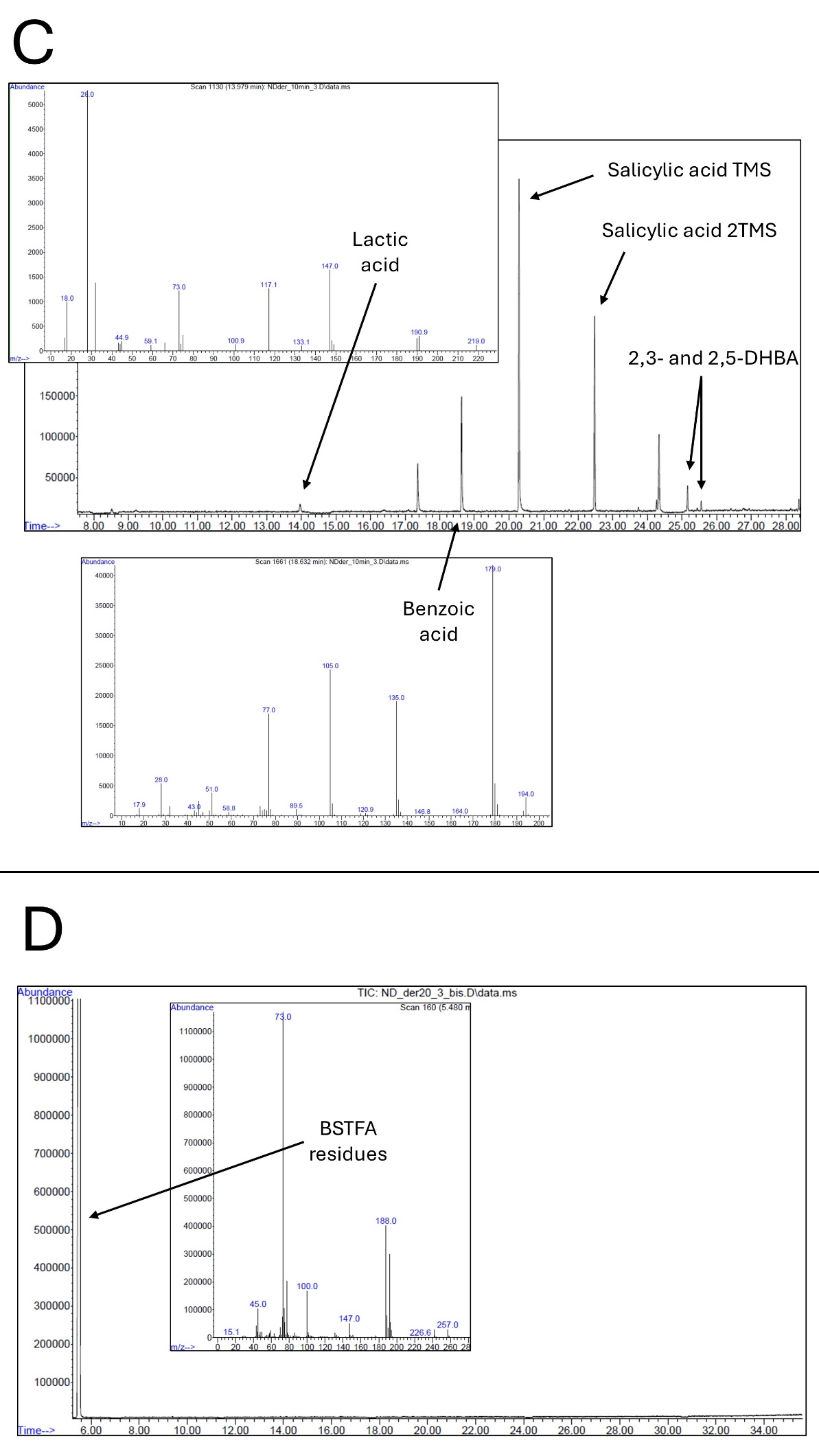 |

**Figure S6.** GC-MS chromatograms of qualitative investigation on byproducts formation. Detection of A) catechol (traces), B) 1-butanol, C) benzoic acid; D) End of treatment

**Figure S7**. OES spectroscopy of HC/ED plasma. ^•^H_β_ and ^•^H_α_ emissions at 486 and 656 nm, respectively.

| 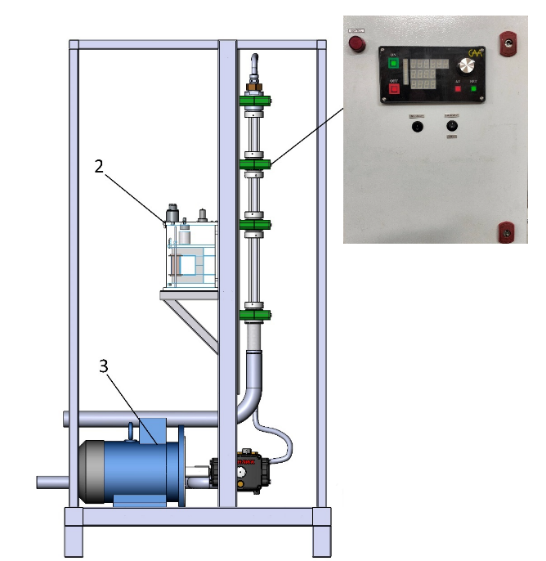 | 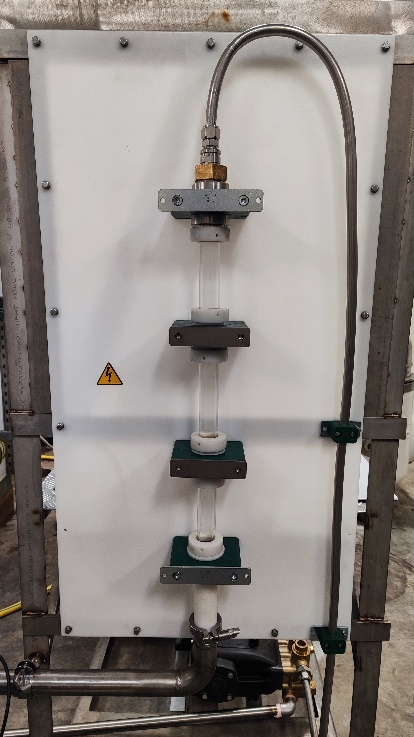 |
| --- | --- |

**Figure S8.** Schematic and pictures of the new semi-industrial scale HC/ED plasma prototype


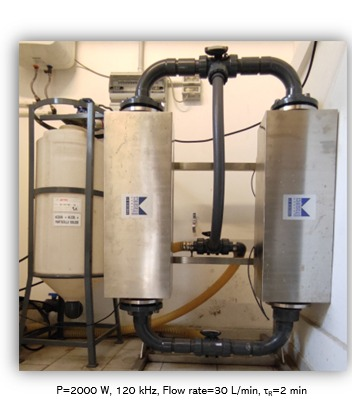


**Figure S9.** Picture of the pilot-scale US unit (BioPush Weber Ultrasonics AG) which operates at a frequency of 120 kHz at a flow rate of 30 L/min.

**Figure S10.** Results of SA degradation under US at a frequency of 120 kHz
